# Cases of Aphonia, Depending upon an Affection of the Head

**Published:** 1832-10

**Authors:** John Webster

**Affiliations:** Physician to St. George's and St. James's Dispensary, &c.


THE LONDON

### Medical and Physical Journal.

404, VOL. LXVIII.] OCTOBER 1832. [76, *New Series*.

"For many fortunate discoveries in medicine, and for the detection of numerous errors, the world is indebted to the rapid circulation of Monthly Journals; and there never existed any work to which the Faculty, in Europe and America, were under deeper obligations than to the 'Medical and Physical Journal of London, now forming a long but an invaluable series."—RUSH.

### ORIGINAL PAPERS.

---

### APHONIA.

*Cases of Aphonia, depending upon an Affection of the Head.*
By JOHN WEBSTER, M.D., Physician to St. George's and St. James's Dispensary, &c.

THE following cases of Aphonia, which, along with some others, have lately come under my observation, as well from the nature of the symptoms exhibited, as the mode of treatment pursued, are, I am disposed to think, worthy of notice; particularly, as the nature and seat of such kind of affections have hitherto been generally viewed in a very different light from what it is intended, in the present paper, to investigate and explain.

In those individuals, there was principally observed a peculiar affection, amounting to an almost total loss of voice, without there being, at the same time, any decided proof of actual disease existing in the larynx or glottis; sufficient, at least, to account for the presence of all the symptoms.

Hitherto similar diseases, or extinctions of voice, as they are sometimes called, have generally been considered by physicians, as likewise by the patients themselves, to depend upon some affection in the larynx, the trachea, or other organs connected with the function of breathing; but from a detail of the symptoms characterizing these four cases, it will be satisfactorily shewn that the loss of voice, in every instance, was occasioned, not by any disease in the organ apparently affected, but in reality to arise from a paralytic state of the nerves distributed on the larynx and glottis; caused, in the first place, by pressure, or some other diseased condition, in the cerebrum.

That affections of the brain, and of the nerves proceeding from that organ, produce disease in distant parts of the human body, is a circumstance almost too well known to require any illustration, since, among other examples, it is seen daily occurring in paralysis, and the like complaints. Nevertheless, to point out how frequently the organs of respiration, and of the voice, are affected by diseases in the cerebrum, and which consequently are sufficient to derange the functions of the nerves proceeding to particular parts of the body, will not be altogether foreign from the present investigation: on the contrary, it appears particularly appropriate, and may serve to explain the point now proposed to be established.

Thus, apoplexy produces stertorous breathing, with a partial, and often a total loss of speech. Hydrocephalus, occurring in children, is frequently accompanied by a croaking kind of sound in the voice, or a spasmodic affection in breathing; and further, in a paper on Hooping-cough I published some years ago in this Journal, it was demonstrated that congestion in the blood-vessels of the brain, and effusion of serum in the ventricles or on the membranes of that organ, which are so commonly observed in the dissection of those dying by this complaint, materially tend, during life, if not to produce, at least to increase in violence and frequency, the fits of coughing and sickness pathognomic of pertussis. Should this opinion be still considered hypothetical, it must, however, have some weight to know that the treatment then recommended, namely, leeches to the forehead and blisters behind the ears, is generally so successful as to cure the patient with certainty and expedition.

Other arguments might be advanced to prove the influence affections of the head have upon the organs of respiration; but it appears almost unnecessary further to explain the action which a diseased condition of the brain and nerves exerts on these organs, and on the voice: more especially since the nervous influence must be the same on these as on other parts of the human body: for, should there exist an affection of that part of the cerebrum, from whence the nerves distributed on the glottis and larynx arise, the functions of that organ must consequently be disordered, or perhaps destroyed: at least, so long as any diseased action, or interruption of function in the nerves, continues: much in the same way as disease of the spine paralyses the bladder, or suspends the motion of an extremity.

Hitherto, reasoning upon such physiological principles has been often overlooked by medical men; and, from inattention to this point, I have been led, in cases similar to those now detailed, totally to neglect the circumstances just mentioned; and, instead of considering complaints, like the present, as depending upon a diseased condition of the brain and nerves, such losses of voice were thought to arise principally, if not wholly, from an affection in the larynx or glottis itself; to which parts, consequently, the remedies formerly employed for their removal were chiefly and, as it was then believed, properly directed.

But the views intended to be explained on this subject will, perhaps, be better understood by considering the history and symptoms of the cases subjoined; especially if we take into consideration the effects of the treatment pursued, in consequence of the above reasoning, which is founded upon the conclusions come to in regard to the actual seat and nature of the complaint; viz. that the loss of voice in these individuals depended upon a paralytic condition of the larynx and glottis, in consequence of derangement of the nervous influence distributed to the parts affected, proceeding from an affection of the brain and nerves; but not, as is commonly supposed, arising from any disease really existing in the larynx or the glottis, as the organ of voice.

Case I. George Wright, æt. sixteen, groom. When first seen, this patient had an attack of bronchitis, which was, however, soon removed by the exhibition of demulcent and aperient medicines, and the application of a blister to the chest: on the 14th of January last, he was considered free from all pectoral complaints.

Nevertheless, at this period an affection or weakness of voice shewed itself; and, on the 16th, it became so remarkable that Wright could only with the greatest difficulty make himself heard by the bystanders, being scarcely able to speak, even in the lowest whisper. Notwithstanding the presence of this symptom, he did not complain of any pain in the throat or chest, nor was there any dyspnœa; but the patient now for the first time mentioned that he had a severe headach, accompanied by drowsiness, and deafness; and, on examining the pupils of both eyes, they were observed to be very much dilated, and almost insensible to the influence of light. It was from these circumstances that attention was especially directed to the cerebral symptoms, which ultimately led me to consider this affection of the head to be, in reality, a principal cause in producing loss of voice in this individual; and subsequent investigation, confirmed by the successful treatment adopted, demonstrated the correctness of the pathological views then entertained.

Five grains of the extract of conium were given at night, followed by an aperient in the morning; and a demulcent medicine was also ordered to be taken, twice or three times a day.

Little or no benefit, as might be expected, followed this mode of treatment: on the contrary, the loss of voice still continued, and it was latterly even so much affected, that the patient sometimes could not articulate a single word; the air during these efforts to speak appearing to pass through the rima glottis, as if he were blowing a musical instrument, without a note being produced. It also merits observation, that if any alleviation of the pain in the head occurred, and especially if, at the same time, the pupils became less dilated, and were sensible to the impression of light, the voice invariably became stronger and more distinct; thus shewing that the condition of the brain, and consequently the function of the nerves distributed on the larynx and glottis, was materially concerned in the disease; and, therefore, it appeared this affection of the head must first be removed, in order to restore the natural tone and strength of the voice.

In accordance with the above reasoning, a blister was applied to each temple on the 24th, which discharged freely; and the patient, when seen on the 26th, was found to have materially improved; the headach was almost gone, excepting over the right eye; the pupils appeared less dilated, and sensible to light, and the words, when speaking, could now be more distinctly articulated, although still in a low tone: the countenance likewise looked clearer, was not so anxious, and, to use the patient's expression, " he felt almost quite well," excepting the continued weakness of his voice.

Extract of conium, which had been taken for the last two nights, was continued, and an aperient mixture prescribed in the morning; at the same time, more nourishment was allowed, as the appetite and digestion had improved. On the 18th, the patient felt considerably better; the pupils contracted when exposed to a strong light, and the deafness and headach were almost removed; whilst the tone of the voice was nearly natural, and he articulated words correctly, although not strongly. A week afterwards, he had almost recovered his usual voice, had no head affection; and, when seen for the last time, in the middle of February, he was convalescent.

Case II. Mrs. Ellis, æt. twenty-one, married, but without children. On the 15th December last, Mrs. E. first consulted me as a patient, when she was affected by hysterical dyspeptic complaints, a slight cold, with an occasional cough; which, nevertheless, became one night so severe, that, in the violence of the attack, some slight streaks of blood were observed in the frothy expectoration; the bowels were costive, and the appetite impaired; but at this date there was not any affection of the head, and the voice was perfectly natural.

Camphor mixture, peppermint water, with aperients, were exhibited; and, after a few days, a blister was applied to the lower part of the chest and epigastrium. By these means the patient's health became much improved, her spirits got better, and, although there was still an occasional cough, she was altogether relieved, and considered to be nearly convalescent. At this period, extract of hyoscyamus was ordered at night, and decoction of bark added to the antispasmodic mixture.

Early in January last, the voice was observed to be weak, but as she was then thought to be only affected with a common hoarseness, very little attention was consequently at first directed to the circumstance. However, on the 12th of the month, the voice became so feeble, that even a single word could with difficulty be articulated; there was no pain of the throat, and she was free from dyspnœa; but she now first complained of being deaf, with a feeling of noise in the ears, and considerable headach, occasionally accompanied by giddiness; and on examining the pupils of both eyes, they were found to be very much dilated, and nearly insensible to light; whilst, on inquiry, it was ascertained that at night she could with difficulty see by the light of a candle, being then, in fact, nearly blind.

At this stage of the disease, an aperient medicine was exhibited in the morning; afterwards decoction of bark with sulphuric æther, and extract of conium at night. By continuing these remedies for a few days, Mrs. Ellis improved a little in her general health; her voice appeared somewhat stronger, but it soon again became as weak as before, and could scarcely at last be heard; the pupils were anew dilated, along with dimness of sight, whilst the noise in the ears, and other head symptoms, already mentioned, again returned. In consequence of these circumstances, on the 2d of February a blister was applied to each temple, and a brisk purgative ordered at night, with a demulcent tonic mixture morning and evening.

The blisters discharged freely; after this, extract of conium and squill were given at night, and an aperient draught in the morning, to be repeated when found necessary. About a week after the application of the blisters, all the head symptoms were nearly removed, and the voice had almost recovered its natural tone and strength; the pupils appeared to be scarcely affected, and the patient could now see to read or work by candlelight; in short, she was considered all but convalescent. On the 12th of February Mrs. Ellis was free from any affection of the voice, and she therefore reported herself quite recovered.

The two following cases occurred some months subsequent to the preceding: they are of a similar character, were successfully treated in the same manner, and they fully confirm the views then entertained regarding the nature of this peculiar affection. The symptoms are related with more brevity, as any lengthened account would be superfluous, and is, indeed, not required to elucidate the subject. One remark, however, will not be here out of place: the two first cases occurred in winter, the latter during the warm weather of summer.

Case III. Dinah Swales, æt. twenty-eight, single; 7th June, 1832. Is of a full habit of body, and has had occasionally hysterical dyspeptic complaints. For the last ten days, has been affected with pain in the head, vertigo, and dimness of sight, at which period she began to lose her voice. At present, she can scarcely articulate so as to be heard, but has no pain of throat or dyspnea. The pupils are dilated, almost totally insensible to light, and she cannot always see distinctly; besides noise in the ears, when flushed by exertion, she feels a throbbing at the temples, whilst the voice totally fails, and she is then almost obliged to express herself by signs. Tongue white; bowels open; pulse soft; skin natural; catamenia regular; feels dyspeptic, and complains occasionally of flatulency. For these complaints, in the first instance, she took compound rhubarb-pill at night, with an aperient demulcent mixture, also containing camphor, morning and evening. Guided by the experience of former cases, on the 9th four leeches were applied to the forehead, and a blister to each temple at night; which remedies acted properly. Next day, there was evidently less dimness of sight, the left pupil was not much dilated, and the noise in the ears and vertigo were considerably diminished. The tone of the voice sounded decidedly stronger, and the articulation of words was more distinct; the appetite was now good, the bowels open, and there scarcely existed any dyspeptic symptoms.

The voice continued daily to improve, as likewise the sight, unless she was heated by exertion, when these symptoms were always observed to become worse. As the patient, in consequence of exposure to cold on the 14th, had a slight cough and expectoration, compound squill pill, with an aperient demulcent mixture, were prescribed, whereby these symptoms were quickly removed. On the 16th, the tone of the voice was improved, the patient felt nearly free from any complaint in the head, and had no affection of the sight, unless she was hurried or fatigued; under which circumstances the voice became decidedly weaker. For these reasons, a second application of blisters to the temples was advised, and the aperient demulcent medicines were continued. When next seen, on the 21st June, the blisters, it was reported, had discharged freely, and the patient was now entirely free from any head symptoms, whilst the sight and voice were perfectly natural; she were in fact convalescent; and it was since ascertained there had been no return of the complaint.

Case IV. Mary Joyce, æt. forty, single; 11th June, 1832. Is of a spare habit of body, and generally in the enjoyment of good health. About a fortnight ago, she began to lose her voice, whilst, at the same time, she complained of pain in the head, giddiness, noise in the ears, and dimness of sight, but felt entirely free from any pectoral complaints, and has not been low-spirited or hysterical. At present there is no dyspnœa, pain in the throat, nor any other symptom indicating an affection of the organs of respiration, excepting that the voice is exceedingly weak, sometimes scarcely audible; she complains, however, of pain in the forehead, over the right eye, along with the other symptoms described as having first appeared a fortnight ago, and the pupils are dilated. Tongue is rather foul; but the bowels are open, and catamenia regular.

Thinking it would be adviseable, in this case, first to try the effect of leeches, without blisters, six were accordingly applied to the centre of the forehead, in the same manner as I formerly recommended, with so decided advantage, in hooping-cough; at the same time, an aloetic and rhubarb pill was ordered night and morning. As the leeches did not bite properly, not much blood was extracted: however, the head next day felt less painful, the sight was improved, and the tone and strength of the voice seemed augmented; nevertheless the pills were ordered to be continued, and six more leeches to be applied, as before, to the forehead.

The second application of leeches was followed by considerable loss of blood; and on the 17th of June all complaint in the head had vanished, whilst the voice was strong, and perfectly natural; so much so, that the patient was discharged a few days afterwards convalescent.

Similar instances of extinction of voice are by no means uncommon; indeed, every medical practitioner must have met with them repeatedly in the course of his professional experience: generally, however, such complaints have been treated by remedies more immediately directed to the throat and parts adjacent, since these structures were apparently the seat of disease. Like other physicians, I have also formerly pursued this plan of treatment, when certainly it proved, in the generality of cases, both tedious and unsatisfactory, unless in individuals where there was actual disease in the larynx or neighbouring parts; then the commonly-followed method of cure should be recommended. But, judging from the details now given, and supported by other examples, which it is unnecessary, and would be tedious to relate, we may fairly conclude little difficulty will henceforward be encountered in treating affections of the voice, wherever any cerebral symptoms accompany the complaint, constituting a peculiarity doubtless of much more frequent occurrence than many are perhaps disposed to suspect, in consequence of similar symptoms being either overlooked, or not thought to be of sufficient importance to merit attention.

To illustrate the great influence affections of the brain have upon the voice, reference might be made to what occasionally occurs in public meetings; where individuals, after considerable exertion in speaking, or from mental excitement, will sometimes lose their voice entirely, and even suddenly, so as to be obliged to stop in the middle of a speech. Intoxication, fear, and strong mental impressions, will likewise deprive the person so affected, sometimes, of the power of articulation. And if such transitory cerebral influences can thus act upon the voice, it will surely, without much hesitation, be granted, that severe affections of the brain, or a more permanent disorder in the functions of the nerves distributed on the larynx and glottis, would produce similar, if not greater, effects.

Anatomically considered, it will be admitted that any affection of the recurrent and internal laryngeal nerves must materially influence the voice; as these nerves constitute the chief agent, or moving power, of that function. And we know, if any tumor press upon the above-named nerves, during their course, from the superior and lateral part of the medulla oblongata, to their distribution on the larynx and parts adjacent; or if these nerves should be cut by accident, or in experiments, the voice then becomes affected, or even destroyed. If such evident causes have so decided an effect upon the voice, other, or more temporary, may likewise produce similar results: at all events, these considerations should not be overlooked, as a paralytic condition, or any disease of these nerves, may as likely exist, as in those of the eye, the taste, or of motion. From these views, the propriety of cupping on the nape of the neck, and the application of blisters there, and to the occiput, in addition to the mode of treatment above detailed, appears evident; and in some cases certainly these means ought to be employed, since undoubtedly they must prove efficient and advantageous.

A late celebrated physician once said, "the brain, and the various diseases to which it is liable, was like a terra incognita, and scarcely understood:" since that time, much has undoubtedly been done by medical inquirers to advance our knowledge of this most important branch of pathology. Still there remains a great deal to be ascertained, so as to be able to clear up some points, that are yet but obscurely explained; and, without wishing to attach more importance to the present subject than it really deserves, it does certainly appear that a diseased condition of the brain, thence affecting the functions of the nerves distributed on particular parts of the body, has more influence in producing complaints like those above described than we are sometimes disposed to allow. Should, therefore, the cases just related, and the few accompanying, although but imperfect observations, prove the means of inducing others to make further inquiry on this subject, so as to confirm more fully, or even to refute, the propositions now advanced; whilst one of the objects had in view, when drawing up this communication, is thereby gained, our knowledge of an interesting class of affections will be, under any circumstances, both improved and extended.

*56, Grosvenor street; September* 1832.

